# Systematic review and meta-analysis of the efficacy of biologic and targeted synthetic therapies in sarcoidosis

**DOI:** 10.1136/thorax-2025-223014

**Published:** 2025-05-19

**Authors:** Katie Bechman, Kathryn Biddle, Aitana Miracle, Kelly He, Mark Gibson, Mark D Russell, Sarah Walsh, Peter Brex, Amit S Patel, Katherine J Myall, Sam Norton, Surinder S Birring, James Galloway

**Affiliations:** 1 Centre for Rheumatic Diseases, King’s College London, London, UK; 2 Department of Dermatology, King’s College Hospital NHS Foundation Trust, London, UK; 3 Department of Neurology, King’s College Hospital NHS Foundation Trust, London, UK; 4 Asthma, Allergy and Lung Biology, King’s College London, London, UK; 5 Department of Respiratory Medicine, King’s College Hospital NHS Foundation Trust, London, UK; 6 Department of Psychology, King’s College London, London, UK

**Keywords:** Sarcoidosis

## Abstract

**Objectives:**

Infliximab, an anti-TNF agent, is used to treat sarcoidosis that does not respond to corticosteroids or second-line agents. The efficacy of other anti-TNF agents, non-TNF biologics and targeted synthetic therapies remains unclear. This study aims to evaluate the role of these therapies in the management of multisystem sarcoidosis.

**Methods:**

We conducted a systematic literature search to identify trials of biological and targeted synthetic therapies in sarcoidosis. Meta-analyses examined %-predicted forced vital capacity (FVC), as mean change from baseline. Heterogeneity was measured using the I^2^ statistic. Vote counting based on the direction of effect, as recommended by the Cochrane network, was used to synthesise study estimates.

**Results:**

The search identified 6777 records. Sixteen studies met the inclusion criteria. These included 8 randomised control trials (RCTs) and 8 single-arm trials. Fourteen studies evaluated biologic therapies: infliximab (n=5), adalimumab (n=2), etanercept (n=2), golimumab (n=1), rituximab (n=1), anakinra (n=1), sarilumab (n=1), ustekinumab (n=1) and efzofitimod (n=1). Two trials assessed the targeted synthetic therapy tofacitinib. Risk of bias was high in five of eight RCTs. Meta-analysis of %-predicted FVC showed modest improvement with treatment (mean change: 4.79% (95% CI 1.22 to 8.35), driven by anti-TNF trials 5.70% (95% CI 1.61 to 9.78). Heterogeneity was substantial (I²=76.3%). In data synthesis using vote counting, infliximab, adalimumab, efzofitimod and tofacitinib demonstrated a positive direction of effect across all estimates, though improvements in several outcomes did not reach thresholds for minimal clinically important differences.

**Conclusions:**

Meta-analysis supports infliximab use in pulmonary sarcoidosis, although improvements in lung function are modest. There is limited but promising evidence for the use of adalimumab and tofacitinib in cutaneous disease and efzofitimob in pulmonary disease. Study interpretation is limited by small sample sizes and heterogeneity in study design and population.

PROSPERO registration number

CRD42024599560

**WHAT IS ALREADY KNOWN ON THIS TOPIC**

**WHAT THIS STUDY ADDS**

- This systematic review and meta-analysis confirms modest efficacy of infliximab for pulmonary sarcoidosis and highlights promising but limited evidence for adalimumab, tofacitinib and efzofitimob in specific disease manifestations.

**HOW THIS STUDY MIGHT AFFECT RESEARCH, PRACTICE OR POLICY**

- The findings underline the need for larger, standardised trials to refine treatment guidelines and investigate the role of non-TNF biologics and targeted synthetic therapies in sarcoidosis management.

## Introduction

 Sarcoidosis is a systemic granulomatous disease characterised by the formation of non-caseating granulomas, which can affect almost any organ. The lungs are involved in 90% of cases. Extrapulmonary manifestations occur in 30%–50% of patients, most commonly affecting the skin, eyes, heart, liver and nervous system.^1^ The disease exhibits significant heterogeneity in clinical presentation, ranging from asymptomatic or self-limiting forms to progressive, multisystem involvement leading to irreversible organ dysfunction and failure.^2^ Although respiratory physicians are often the first point of contact, optimal management necessitates a cross-speciality approach involving rheumatologists, cardiologists, ophthalmologists and neurologists.

Sarcoidosis imposes substantial morbidity burden, with symptoms such as dyspnoea, fatigue and chronic pain significantly impairing health-related quality of life. The disease course is unpredictable, and while spontaneous remission occurs in some cases, chronic or progressive disease develops in a substantial proportion of patients. Mortality is estimated at approximately 5%, primarily attributable to respiratory failure and cardiac involvement.^3^ Although often considered a rare disease, sarcoidosis is the most prevalent cause of fibrotic lung interstitial disease after idiopathic pulmonary fibrosis^4^ and the second most common chronic respiratory condition in young adults after asthma.^5^ It is also recognised that a substantial proportion of sarcoidosis cases remains undiagnosed.

Despite its clinical importance, there is a paucity of research into sarcoidosis. Clinical trials are limited by the perceived rarity of the disease, its heterogeneous presentation and the absence of standardised outcome measures.^6^ Consequently, treatment strategies remain non-standardised, with guidelines primarily based on small, uncontrolled trials and expert opinion^3 7^

Corticosteroids are widely accepted as first-line therapy across multiple treatment algorithms.^6^,^9^ While short-term benefits are well documented, their impact on disease progression and long-term mortality remains unclear.^10^ To minimise steroid toxicity, second-line immunosuppressive agents, such as methotrexate, mycophenolate, leflunomide and azathioprine are frequently used in clinical practice^3 6 8 9^; however, these agents are not always effective or well tolerated.^11 12^ TNF inhibitors, particularly infliximab and adalimumab, are considered third-line options.^3 6 8 9^ In England, infliximab has been approved for use in refractory disease that has failed to respond to corticosteroids and a second-line agent, with access available through routine commissioning.^13^ Beyond TNF inhibitors, there is increasing interest in non-TNF biologics and targeted synthetic therapies, including Janus kinase (JAK) inhibitors, which have shown promise in case series and small studies,^14^ yet are largely excluded from previous systematic reviews.^15 16^ Given the pressing need for effective and personalised treatment strategies, a comprehensive evaluation of biologic and targeted synthetic therapies is essential. This systematic review and meta-analysis will assess the efficacy of biologic and targeted synthetic therapies in sarcoidosis, strengthening the evidence base to guide clinical practice and future guidelines.

## Method

This study was conducted in accordance with the Preferred Reporting Items for Systematic Reviews and Meta-Analyses guidelines and registered with the International Prospective Register of Systematic Reviews (registration number CRD42024599560).

### Data sources and search strategy

A systematic literature search was performed by two investigators (KH and AM) using MEDLINE, EMBASE and ClinicalTrials.gov from database inception to October 2023. The search was rerun until June 2024. The drugs of interest were anti-TNF biologics: infliximab, adalimumab, golimumab, certolizumab and etanercept; non-TNF biologics: abatacept, tocilizumab, sarilumab, secukinumab, ixekizumab, brodalumab, bimekizumab, ustekinumab, guselkumab, risankizumab, tildrakizumab, rituximab, belimumab, omalizumab canakinumab and anakinra; GM-CSF inhibitor: namilumab; neuropilin-2 inhibitor: efzofitimod; and JAK inhibitors: tofacitinib, baricitinib, upadacitinib, filgotinib, abrocitinib and ruxolitinib. The reference lists of published systematic reviews and the clinicaltrials.gov website were manually searched to ensure that additional pertinent studies were not missed.

### Study selection

We identified English language publications of cohort studies and clinical trials. Single-arm studies were deemed eligible for inclusion if the study (1) enrolled adult patients with sarcoidosis and (2) examined biologics or targeted synthetic therapies. Comparator trials were eligible for inclusion if they met the same two criteria, while also including a placebo comparator or another active treatment arm. Conference abstracts, case reports and case series were excluded, as were studies that presented duplicate data or insufficient data on treatment efficacy. Titles and abstracts of studies retrieved using the search strategy were screened independently by two investigators, KH and AM. The full text of the potential studies for inclusion was retrieved and assessed for eligibility. Study quality and risk of bias were assessed using the Cochrane Collaboration’s Risk of Bias 2 tool for comparator arm trials and the Newcastle Ottawa Scale for single-arm cohort studies.

### Data collection

Data were extracted independently by two reviewers. Disagreements over study eligibility or risk of bias were resolved through discussion with a third reviewer (KB). Data collected included the source (author, journal and publication date), study design, sample size, dosage and schedule of the treatment, duration of study, patient characteristics, organ involvement, concomitant therapies and primary and secondary trial outcomes. Studies with incomplete data were identified, and requests to provide missing data were made by contacting study authors.

### Data synthesis and statistical analysis

Our primary objective was to conduct a meta-analysis across multiple outcomes, including lung function, cutaneous activity scores, the Extrapulmonary Physician Organ Severity Tool (ePOST) and patient-reported outcome (PRO) measures. However, given the heterogeneity of studies, including clinical and methodological diversity and differences in effect measures, we were limited in our ability to meta-analyse the data. We therefore used a method of vote counting based on the direction of effect to summarise and synthesise estimates, a technique that is recommended by the Cochrane network.^17^ Each effect estimate was categorised as showing benefit or no benefit based on the observed direction of effect alone. Statistical significance was not considered in the categorisation. The size of the effect was only considered if the effect showed benefit. In this situation, if the improvement was less than the minimal clinically important difference (MCID), the effect would be categorised as ‘of uncertain benefit’. Where a proportion of patients was reported rather than an effect size, this was categorised as showing benefit if more than half met the outcome of interest. The number of effects showing benefit was compared with the number of effects showing uncertain or no benefit and tabulated using a traffic light matrix.

A meta-analysis of %-predicted FVC was conducted, as it was the only outcome across both single-arm and comparator arm studies with consistent effect estimates. While %-predicted FVC is a recognised primary endpoint in pulmonary fibrosis trials, its clinical relevance in non-fibrotic pulmonary sarcoidosis is limited, as lung function is often normal and FVC does not reliably correlate with disease activity or symptom burden.^7^ In this meta-analysis, improvement in %-predicted FVC was defined as a numerical increase from baseline in the treatment arm and reported as a mean value with 95% CIs. If studies presented mean values with SD, these were converted to mean value with 95% CIs for consistency. A pooled estimate was calculated using a random-effects model with the restricted maximum likelihood method and presented in a forest plot. Between-study heterogeneity was assessed using Cochran’s Q test, I² statistic, and τ², which quantifies the variance of true effect sizes across studies. Sensitivity analyses were performed, including a leave-one-out analysis and a meta-analysis restricted to studies at low to moderate risk of bias. Publication bias was explored using funnel plots and Egger’s test for funnel asymmetry. Analyses were conducted using Stata 18.

## Results

### Search results and study characteristics

**Figure 1:**
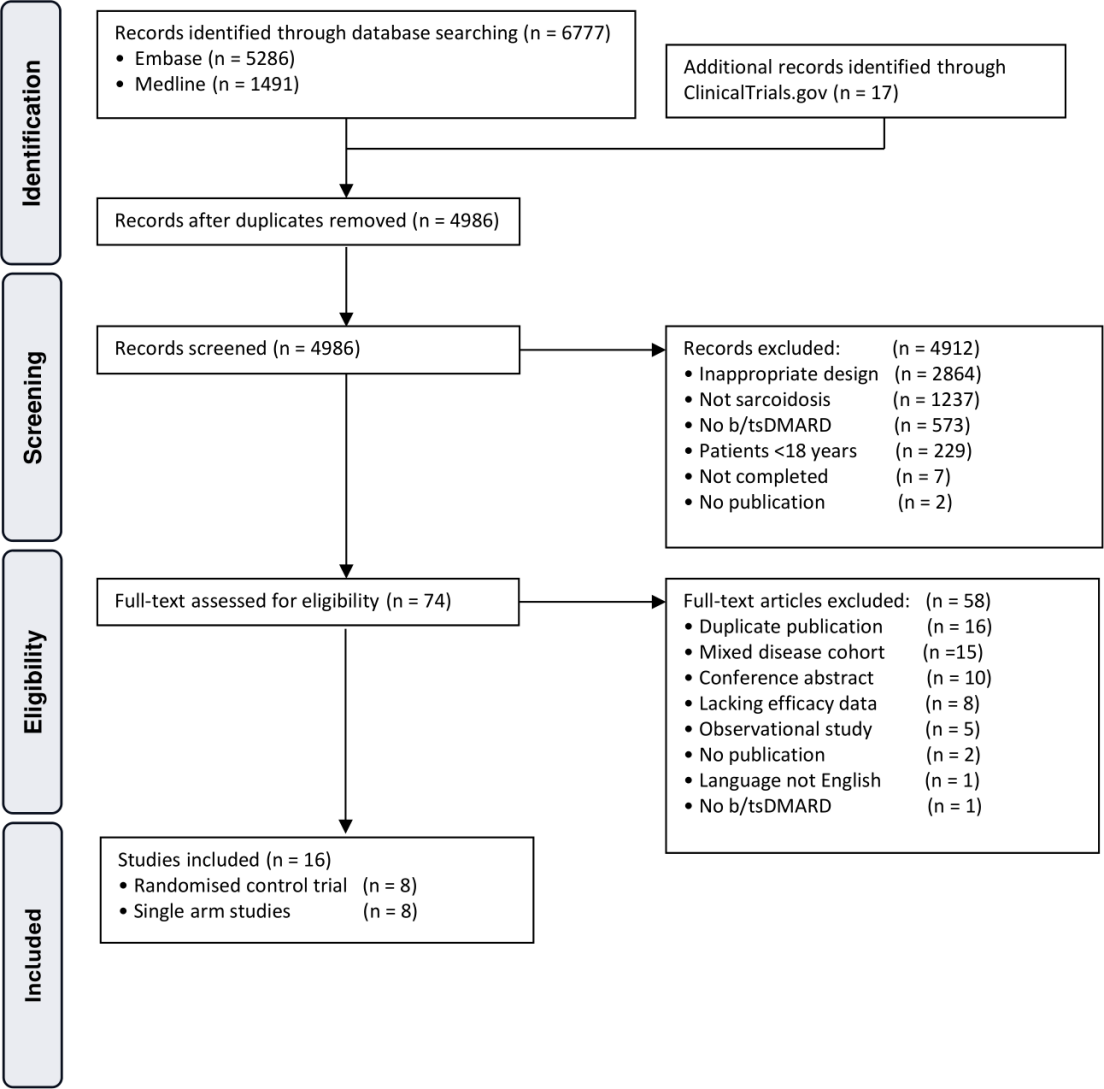
Flowchart of systematic search for studies reporting the efficacy of biologics or targeted synthetic therapies in the treatment of sarcoidosis.

We identified 6777 records, from which 4986 titles and abstracts were screened, and the full texts of 74 articles were assessed for eligibility (figure 1). Sixteen publications (of 15 trials) met inclusion criteria. This included seven randomised control trials (RCTs), one of which was analysed in two separate publications with different disease outcomes^18 19^ and eight single-arm studies.

Six RCTs featured a two-arm design with a placebo comparator; one study had three arms (golimumab, ustekinumab and placebo)^20^ (table 1 and online supplemental file 1). Biological therapies were assessed in six anti-TNF trial arms (n=273) of which infliximab was the most frequently investigated (three trials, n=199), and three non-TNF arms (n=92) including ustekinumab, anakinra and efzofitimod. The median sample size overall was 19 (IQR: 16 to 138) and the median study period was 16 weeks (IQR: 12–24). Of the eight single-armed studies, seven were open-label prospective trials and one was an open-label prospective trial, followed by a randomised drug withdrawal (table 1). Anti-TNF therapy was assessed in three trials (n=97), of which infliximab was the most investigated agent (three trials, n=69). There were two non-TNF studies: rituximab (n=10) and sarilumab (n=15) and two targeted synthetic therapies studies: tofacitinib (n=15). The median sample size was 12 (IQR: 10 to 16) and the median study period was 26 weeks (IQR 21 to 52).

**Table 1:** Summary of included comparator arm studies

Study	Drug	Number on drug	Dose	Age	Female (%)	Concomitant medications	Predicted FVC	Primary outcome
**Randomised control trials**								
*Culver et al* ^30^	Efzofitimod	9	5 mg/kg	50.8±9.8	55.6	CS (100), MTX (33.3), AZA (11.1)	83.8±16.6	TEAE-free survival at week 24
	Efzofitimod	8	3 mg/kg	51.8±11.4	50	CS (100), LEF (12.5)	83.8±7.3	
	Efzofitimod	8	1 mg/kg	54.5±11.3	50	CS (100), MTX (25), HCQ (12.5)	68.3±9.7	
	Placebo	12	NA	52.5±10.2	58.3	CS (100), MTX (33.3), AZA (16.7)	77.3±11.5	
*Judson et al* ^20^	Ustekinumab	60	180 mg ->90 mg	49.8±10.2	48.3	CS (76.7), MTX (20), AZA (1.7)	64.3±NR	Change at week 16 in % predicted FVC
	Golimumab	55	200 mg ->100 mg	50.0±9.4	49.1	CS (80), MTX (21.8), AZA (7.3)	68.0±NR	
	Placebo	58	NA	49.5±9.5	50	CS (70.7), MTX (18.9), AZA (5.2)	68.4±NR	
*Pariser et al* ^21^	Adalimumab	10	80 mg ->40 mg	46.0±NR	80	NR	NR	PGA score of 2 or less
	Placebo	5	NA	52.6±NR	80	NR	NR	
*Baughman et al* ^18^	Infliximab	47	5 mg/kg	46.5±8.7	40.4	CS only (51.1), csDMARD only (8.5), CS+csDMARD (40.4)	69.5±8.6	Change at week 24 in % predicted FVC
	infliximab	46	3 mg/kg	49.3±9.4	47.8	CS only (43.5), csDMARD only (8.7), CS+csDMARD (47.8)	67.7±9.6	
	Placebo	45	NA	45.3±9.4	42.2	CS only (57.8), csDMARD only (4.4), CS+csDMARD (37.8)	68.8±11.1	
*Judson et al* ^19^	Infliximab	93	3 mg/kg or 5 mg/kg	47.8±9.1	44.1	NR	68.6±9.1	Change at week 24 in ePOST
	Placebo	45	NA	45.3±9.4	42.2	NR	68.8±11.1	
*Baughman et al* ^28^	Etanercept	9	25 mg	NR	88.9	CS (55.6), MTX (100), AZA (0)	68±NR	Ophthalmologist exam at 6 m
	Placebo	9	NA	NR	100	CS (22.2), MTX (100), AZA (0)	92±NR	
*Rossman et al* ^26^	Infliximab	13	5 mg/kg	46.77±2.31	61.5	CS (69.2), MTX (NR), AZA (NR)	50.6±4.4	Change at week six in % predicted FVC
	Placebo	6	NA	49.33±4.92	16.7	CS (66.7), MTX (NR), AZA (NR)	56.8±5.2	
*Kron et al* ^22^	Anakinra+SOC	7	100 mg per day	NR	NR	NR	NR	Change in hs-CRP at 28 days
	SOC	9	NR	NR	NR	NR	NR	SOC
**Single arm studies**								
*Friedman et al* ^32^	Tofacitinib		5 mg BD	40.8±NR	20	CS (100), MTX (NR), AZA (NR)	86.6±NR	≥ 50% reduction in CS at week 16
*Damsky et al* ^24^	Tofacitinib		5 mg BD	56±NR	40	CS (50), MTX (40), AZA (0), HCQ (10)	NR	Change in CSAMI activity score at 6 months
*Utz et al* ^29^	Etanercept		25 mg twice weekly	49.4±10.7	58.8	NR	91.1±16.5	NA
*Sweiss et al* ^27^	Adalimumab		40 mg weekly	45.3±12.7	90.9	CS (45.5), MTX (36.4), AZA (9.1), LEF (9.1), MMF (36.4), CYC (9.1)	61±12	Change from baseline to week 24 in % predicted FVC
*Kullberg et al., 2020*	Infliximab		3–5 mg	47.6±5.1	15.4	CS (92), MTX (16.7), AZA (0)	70±NR	NA
*Vorselaars et al* ^25^	Infliximab		5 mg/kg	48.7±10.1	35.7	CS (42.9), MTX (82.1), AZA (7.1), LEF (1.8)	78.8±NR	NA
*Sweiss et al* ^31^	Rituximab		1 g weeks 0 and 2	49±NR†	30	NR	57.3±14.2	Safety
*Baker et al* ‡ ^23^	Sarilumab		200 mg	57.0±NR†	20	CS (100), DMARDs (33.3)	92.0±NR†	Flare-free survival on CS taper. Flare was defined as the need for rescue, significant worsening of disease or cessation of study intervention.

* Patients told to stop MTX at study inclusion.

† Median and not mean.

‡ Only includes RCT period of trial.

§ Analysis of Baughman *et al*.^18^

AZA, azathioprine; DMARDs, Disease-Modifying Anti-Rheumatic Drugs; MTX, methotrexate; NA, not assessed; NR, not reported; % predicted FVC, percentage of predicted forced vital capacity’s, corticosteroids; RCT, randomised controlled trial; SOC, standard of care.

Six comparator arm studies included extrapulmonary endpoints. Two studies included an extrapulmonary primary endpoint: one enrolled patients with cutaneous sarcoidosis and defined the primary outcomes as the Physician Global Assessment of the overall volume of cutaneous lesions relative to the baseline^21^; the other enrolled patients with cardiac sarcoidosis and defined the primary endpoint as change in C reactive protein, with secondary endpoints including change in left ventricular (LV) FDG-PET-CT uptake and LV ejection fraction.^22^ Four comparator studies included extrapulmonary secondary endpoints: two examined ePOST^19 23^ and two examined cutaneous endpoints.^18 20^ Of the single-arm trials, one study enrolled patients with cutaneous sarcoidosis and included CSAMI as the primary outcome,^24^ and one enrolled participants with cutaneous and neurosarcoidosis and included a descriptive analysis of organ function as the primary outcome measure, including descriptions of skin and sensation improvement, serum inflammatory markers and FDG-PET-CT activity.^25^

### Risk of bias

The risk of bias was high in five of the eight comparator arm trials. Two studies had a moderate risk of bias and one study had a low risk of bias. All seven single-arm trials were at high risk of bias in at least one criterion (online supplemental figure 2).

### Anti-TNF biologics

**Figure 2:**
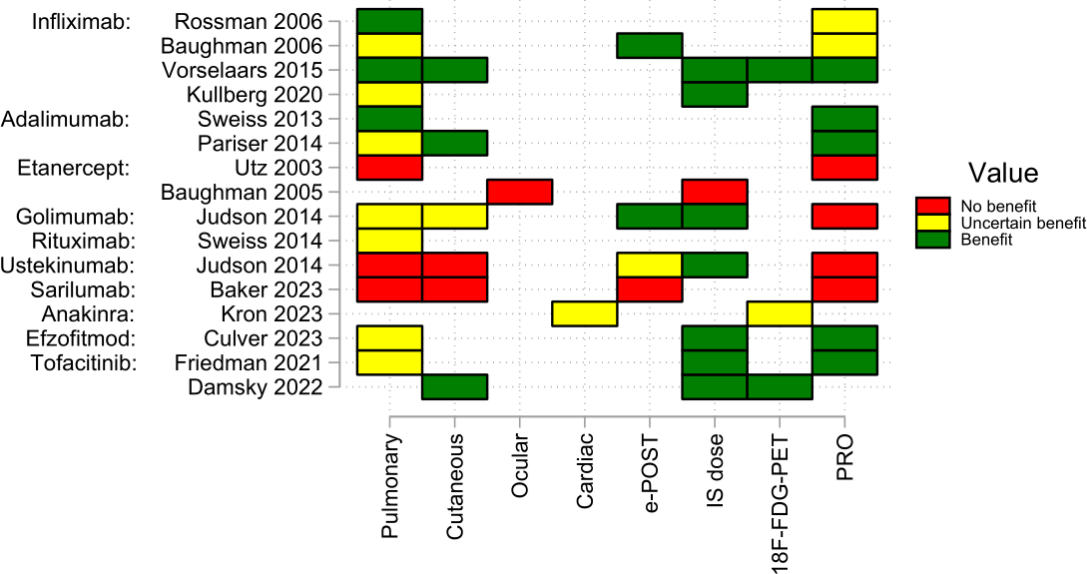
Vote counting data synthesis based on the direction of effect across outcomes, presented in traffic light matrix by drug, trial and outcome. Effect estimate categorised as showing benefit (green) or no benefit (red) based on the observed direction of effect alone. The size of the effect was only considered if the effect showed benefit. In this situation, if the improvement was less than the MCID, the effect would be categorised as ‘of uncertain benefit’ (yellow). Where a proportion of patients was reported rather than an effect size, this was categorised as showing benefit if more than half met the outcome of interest. MCID, minimal clinically important difference.

Data from two RCTs and two single-arm trials suggest that infliximab is efficacious in pulmonary sarcoidosis, with less support for cutaneous disease or patient-reported outcomes (figure 2 and online supplemental file 3). Only one RCT met its primary outcome, with a statistically significant improvement in %-predicted FVC.^18^ Non-significant improvements in 6 min walk distance, Borg’s dyspnoea score and radiological changes were demonstrated in both RCTs.^26^ The single-arm trials also confirmed improvements in pulmonary function tests. In extrapulmonary disease, statistically significant improvements were seen in the ePOST.^19^ Evidence was less consistent in cutaneous disease; although a single-arm trial demonstrated improvement in skin lesions.^25^

Adalimumab was evaluated in one RCT and one single-arm study. In the single-arm study, one-third of patients demonstrated ≥5% improvement in %-predicted FVC^27^ (figure 2 and online supplemental file 3). However, in the RCT, there was no significant improvement in pulmonary function when compared with placebo. In contrast, there were statistically significant improvements in cutaneous disease (target lesion area and volume), although changes in Dermatology Life Quality Index and Sarcoidosis Health Questionnaire scores did not meet statistical significance.^21^

Golimumab was examined in one RCT, which also evaluated the IL12/23 inhibitor ustekinumab. Neither biologic demonstrated a statistically significant improvement in pulmonary function tests (figure 2 and online supplemental file 3). Non-significant improvements in cutaneous disease, ePOST score and corticosteroid dose reduction were observed with golimumab.^20^ Etanercept was investigated in two studies; these were both negative trials. In the RCT, etanercept associated with deteriorating ocular disease^28^ and in the single-arm study in pulmonary disease were terminated prematurely due to an excess of treatment failures.^29^

### Non-TNF biologics

As previously described, ustekinumab was not effective in pulmonary or cutaneous disease.^20^ Anakinra, an IL-1 inhibitor, was assessed in one RCT in cardiac sarcoid. In this trial, the primary endpoint of improvement in CRP at 28 days was met; however, there was no statistically significant improvement in LV ejection fraction or LV uptake on FDG PET^22^ (figure 2 and online supplemental file 3). Efzofitimod, a novel biologic that targets neuropilin 2 receptor protein, was investigated in one RCT in pulmonary sarcoidosis. Improvements in pulmonary function, corticosteroid reduction and PROs were demonstrated, with a dose-dependent effect, although these findings did not achieve statistical significance.^30^ The IL-6 inhibitor receptor, sarilumab, was evaluated in a single-arm study in pulmonary and extrapulmonary disease.^23^ This was a negative trial with a deterioration in lung function and CT chest findings. Rituximab was studied in a single-arm trial in pulmonary sarcoidosis. Clinical response was inconsistent among patients, with no statistically significant improvement in FVC. Two patients died during the study period due to progression of disease.^31^

### Targeted synthetics

The only targeted synthetic to be investigated in sarcoidosis is a JAK inhibitor, with two single-arm trials evaluating tofacitinib. In cutaneous sarcoidosis, all 10 patients experienced improvement in their skin, with 6 patients showing a complete response. Improvements in PET-CT uptake were also noted (figure 2 and online supplemental file 3).^24^ In five patients with pulmonary involvement on imaging, three fully tapered corticosteroids with no worsening in pulmonary function. One patient was withdrawn due to worsening neurologic sarcoidosis, though their pulmonary disease remained well-controlled.^32^

### Data synthesis: vote counting based on the direction of effect

All 15 studies were included in the vote counting on direction of effect. These were defined by drug and outcome. Outcomes were grouped into (1) pulmonary, (2) extrapulmonary (cutaneous, cardiac, ocular and ePOST score), (3) immunosuppression, (4) PROs and (5) PET-CT. Effect estimates were presented alongside p values, although statistical significance was not considered in the categorisation of benefit. Both etanercept and sarilumab were categorised as not showing benefit across all outcomes. In contrast, infliximab, adalimumab, efzofitimob and tofacitinib exhibited benefit with positive direction of effects for all estimates. However, in 25% of these, the improvement of the estimate was less than the MCID and the effect was categorised as ‘of uncertain benefit’ (figure 2 and online supplemental file 3).

### Pairwise meta-analysis of improvement in %-predicted FVC in RCTs or single-arm studies

Ten studies reported numerical improvement in %-predicted FVC from baseline, including five RCTs and five single-arm studies, with a median study duration of 24 weeks (IQR 16–24) (online supplemental file 4). Two studies could not be included in the meta-analysis due to insufficient published data.^23 32^

**Figure 3:**
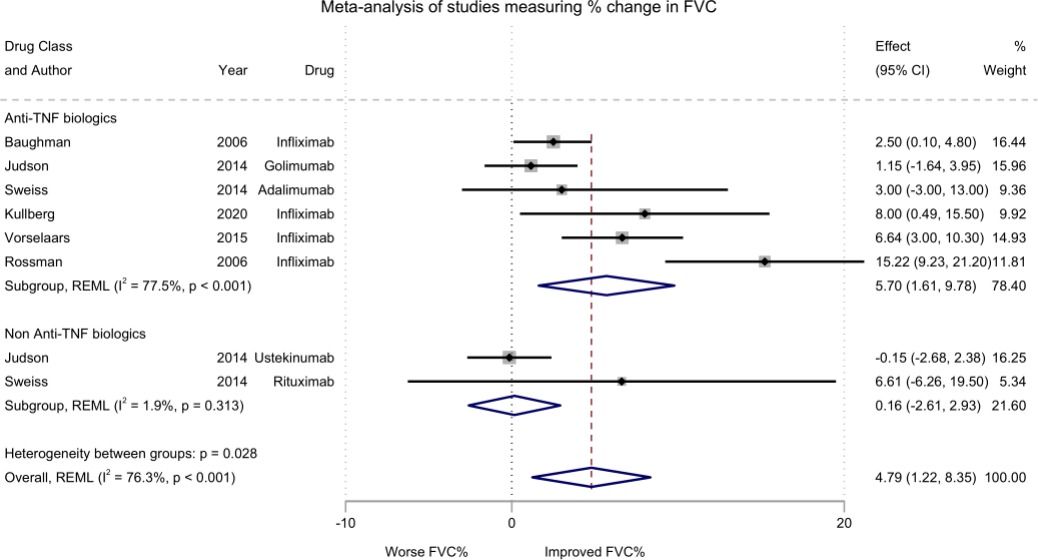
Forest plot presenting pairwise meta-analysis of improvement in % predicted FVC in RCTs or single-arm studies, ES=change% predicted FVC. FVC, forced vital capacity; RCT, randomised controlled trial. NOTE: Weights and between-subgroup heterogeneity test are from random-effects model

The pooled mean change in %-predicted FVC across all included studies was 4.79% (95% CI 1.22 to 8.35) (figure 3). This modest improvement in lung function suggests a potential benefit; however, the relatively wide CI reflects uncertainty around the estimate. Subgroup analysis by drug class showed a pooled mean change of 5.70% (95% CI 1.61 to 9.78) for anti-TNF biologics, compared with 0.16% (95% CI −2.61 to 2.93) for non-TNF biologics. This suggests the apparent benefit is driven primarily by anti-TNF agents, while non-TNF biologics showed no clear evidence of benefit. The overall meta-analysis demonstrated substantial heterogeneity (Cochran’s Q=29.6, p<0.001, I² = 76.3%) with a high between-study variance (τ²=18.7). When stratified by drug class, heterogeneity was particularly pronounced among anti-TNF biologics (Q=22.2, p<0.001, I²=77.5%). This degree of heterogeneity reduces certainty around the true effect size. In contrast, studies on non-TNF biologics exhibited low heterogeneity (Q=1.02, p=0.313, I²=1.9%).

In the leave-one-out analysis, the effect estimates remained within the overall range of the primary meta-analysis, supporting the robustness of the pooled estimate. CIs remained stable, with no single study exerting extreme dominance. The largest reduction in effect size occurred when Rossman *et al*^26^ was omitted, lowering the pooled estimate from 4.79% to 2.91%, highlighting its influence. Similar findings were observed when restricting the analysis to anti-TNF studies (online supplemental file 5).

In sensitivity analyses restricted to studies with low to moderate risk of bias (n=3), the pooled mean change in %-predicted FVC was 3.04% (95% CI 0.75 to 5.33), which was lower than the primary pooled estimate. This suggests that the overall findings may be partially influenced by studies at high risk of bias. However, heterogeneity was substantially reduced (Cochran’s Q=1.88, p=0.394, I²=0.0%), indicating less between-study variability, suggesting that study quality may contribute to the observed heterogeneity (online supplemental file 6).

Funnel plots suggested the potential presence of small study effects, raising the possibility of publication bias or heterogeneity due to differences in study precision. Although visual asymmetry was noted, Egger’s test did not detect statistically significant asymmetry (p=0.15). This result should be interpreted with caution given the limited power of Egger’s test in meta-analyses with few studies (online supplemental file 7).

## Discussion

We conducted the first comprehensive systematic review and meta-analysis to include biologics beyond anti-TNFs, as well as targeted synthetic JAK inhibitors, in sarcoidosis. We identified 16 publications (reporting on 15 trials) covering a wide range of therapeutic agents. Only seven of these trials were RCTs, most of which were published over a decade ago. Due to the heterogeneity of studies and variability in reported effect measures, change in %-predicted FVC was the only outcome suitable for meta-analysis. We observed an improvement in %-predicted FVC, driven primarily by anti-TNFs. Although the effect was small, it fell within the MCID range of 2%–6% derived from studies in idiopathic pulmonary fibrosis and scleroderma.^33 34^ Our data synthesis provided some evidence of beneficial effects across both pulmonary and extrapulmonary sarcoidosis with infliximab, adalimumab, efzofitimod and tofacitinib. However, improvements in several outcomes were modest, often failing to meet the MCID threshold, and the trials were limited by small sample sizes. Evidence for other anti-TNFs and non-TNF biologics was less encouraging.

The efficacy of infliximab and adalimumab is biologically plausible given the central role of TNF-α in the pathogenesis of sarcoidosis. Since many of the cytokines implicated in sarcoidosis progression signal via the JAK-STAT pathway, there is also a strong rationale for the potential effectiveness of tofacitinib.^33^ Efzofitimod offers an alternative mechanism of action by targeting neuropilin-2, a receptor expressed on immune cells such as alveolar macrophages, which play a key role in granuloma formation in sarcoidosis.^30^ In contrast, the lack of efficacy observed with etanercept may reflect its lower affinity for transmembrane TNF-α compared with anti-TNF monoclonal antibodies.^34^ This partial inhibition could allow redistribution of TNF-α to sites of higher concentration, such as the lungs and lymphatic system, ultimately contributing to therapeutic failure.^34^ Similarly, rituximab depletes CD20-expressing B cells, a strategy effective in B cell-mediated diseases like rheumatoid arthritis. However, sarcoidosis is predominantly driven by type 1 helper T cell responses, with minimal B cell involvement, making B cell depletion an unlikely therapeutic target. Sarilumab, which blocks IL-6 signalling, also lacks efficacy, suggesting IL-6 is not a key cytokine in sarcoidosis-related inflammation.

Previously published systematic reviews of biological therapies in sarcoidosis^15 16 35^ have been limited to anti-TNF therapies. These reviews report a beneficial effect of infliximab in both pulmonary and extrapulmonary disease, with possible efficacy for adalimumab.^16^ Two of these reviews included meta-analyses assessing changes in FVC, although these analyses were constrained by the small number of trials and limited sample sizes.^16 35^

This systematic review highlights the wide variation in outcome measures used in sarcoidosis trials, many of which fail to capture the diverse multisystem manifestations of the disease. Although FVC is frequently used, it is not an ideal endpoint for assessing respiratory involvement. It correlates poorly with symptom burden, and as disease trajectories in sarcoidosis can vary significantly, for some patients, maintaining FVC stability may represent a positive outcome. In fibrotic disease, FVC is unlikely to improve significantly in response to treatment. These patients are often more refractory to conventional immunosuppressive therapies and may be over-represented in clinical trials due to their progressive disease course and higher symptom burden. The limited improvement in FVC observed in some studies may reflect the inclusion of a greater proportion of patients with fibrotic disease rather than a true lack of treatment efficacy. PROs are increasingly used to capture the patient’s perspective on disease burden, functional status and quality of life. While these tools are valuable,^36 37^ there is no universally agreed-upon set of PROs for sarcoidosis, leading to inconsistencies in how data are compared across trials. Furthermore, PROs may not fully capture symptoms across organ systems and can lack sensitivity to detect small but clinically meaningful changes, particularly in refractory disease, where stabilisation may be considered a success. Extrapulmonary involvement remains under-represented in trials, and despite neurosarcoidosis forming the basis for infliximab’s initial licensing in sarcoidosis in the UK, it featured in only one clinical trial.

This systematic review and meta-analysis has several notable strengths. It was conducted using a comprehensive and systematic literature search, designed to capture all relevant studies across multiple databases and sources. The search strategy was developed and executed according to a prespecified protocol, ensuring transparency and reproducibility. Study selection, data extraction and quality assessment followed standardised methods and predefined criteria to minimise bias. A key challenge was the considerable methodological heterogeneity across the included studies, particularly in relation to outcome measures, analytical approaches and reporting standards. This variability precluded conventional meta-analysis for many outcomes. To synthesise these diverse data, we applied vote counting based on the direction of effect, a method recommended when statistical pooling is not feasible. Although this approach does not generate pooled effect estimates, it offers a transparent summary of the consistency and direction of effects across studies, providing valuable insight into overall trends in the literature.

However, there are several important limitations to our study. Inconsistent endpoint reporting across trials limited the feasibility of additional quantitative synthesis. During data extraction, we observed that several clinically important outcomes, including extrapulmonary disease severity scores, cutaneous disease measures, PROs and glucocorticoid reduction, were inconsistently reported and often lacked essential statistical detail, such as measures of variance, precluding meta-analysis. By using vote counting, we were able to summarise findings across this broader range of outcomes; however, this method only captures qualitative signals and trends, rather than precise effect estimates. This highlights the urgent need for greater standardisation in outcome selection, measurement and reporting in future sarcoidosis trials, to enable more robust evidence synthesis and better inform clinical guidelines.

Where meta-analyses were performed, they relied on %-predicted FVC as the primary quantitative outcome. This reflects a pragmatic choice driven by the availability and consistency of reported data, rather than an assumption that FVC is the most clinically meaningful endpoint in sarcoidosis. Although sarcoidosis has traditionally been described as a restrictive lung disease, fewer than half of patients with abnormal lung function demonstrate a restrictive phenotype on pulmonary function testing.^12^ This raises concerns that trials relying solely on FVC as the primary endpoint risk misclassifying potentially effective therapies as ineffective, particularly for patients with disease phenotypes less likely to show measurable improvements in FVC. In addition, there was considerable variation in how FVC was reported across studies, with differences in units and frequent omission of key statistical parameters such as SD or SE. In the meta-analyses examining FVC, the I² statistic was high, indicating substantial heterogeneity and uncertainty around the pooled estimates. This variability likely reflects differences in study design (eg, follow-up duration, dosing regimens) and population characteristics, complicating the interpretation of pooled results. Another important limitation relates to the potential for publication bias. Although visual inspection of the funnel plot raised some concern, suggesting possible asymmetry indicative of publication bias, this was not statistically confirmed by Egger’s test. The absence of clear statistical evidence, however, does not entirely exclude the possibility of bias, particularly given the relatively small number of included studies. Finally, high risk of bias was identified in one or more domains of five RCTs and seven single-arm studies. Given the limited number of trials eligible for inclusion in the meta-analyses, these studies were retained to maximise the available evidence. To account for this, sensitivity analyses were performed, including leave-one-out analyses and exclusion of high risk-of-bias studies, which demonstrated consistent findings.

In conclusion, we observed a modest improvement in FVC, largely driven by anti-TNF therapies, with changes falling within the MCID range derived from other fibrotic lung diseases. Infliximab has the strongest evidence supporting its use, with benefits seen in both pulmonary and extrapulmonary sarcoidosis. Based on the current evidence, and in keeping with guidelines, infliximab should be considered for patients with refractory sarcoidosis, particularly those with severe pulmonary disease or organ-threatening extrapulmonary involvement, after failure of corticosteroids and conventional immunosuppressants. Adalimumab may be a reasonable alternative if infliximab is poorly tolerated, contraindicated or inaccessible, particularly for patients requiring long-term therapy where self-administration offers a practical advantage. For tofacitinib and efzofitimod, emerging data suggest potential benefit, particularly for patients with multiorgan disease or those intolerant to anti-TNFs, but their use should currently be limited to research settings or in highly selected refractory cases after careful multidisciplinary discussion. Larger, well-designed trials are needed to clarify the role of these therapies.

A major challenge in advancing sarcoidosis treatment is disease heterogeneity, which complicates clinical trial design and patient selection. To improve trial efficiency, future research should focus on refining disease phenotypes to enable the recruitment of more homogeneous patient cohorts. Additionally, there is a critical need for the development and validation of standardised trial endpoints that accurately reflect disease activity and treatment response. Currently, biological therapies are typically reserved for patients who have failed corticosteroids and DMARDs. In other inflammatory diseases, such as rheumatoid arthritis and inflammatory bowel disease, early treatment escalation improves outcomes. However, in sarcoidosis, the optimal timing for initiating and escalating therapy, as well as the patient populations most likely to benefit, remains unclear. Addressing these research priorities will guide more efficacious clinical trials in sarcoidosis and drive more effective treatment strategies.

## Supplementary material

10.1136/thorax-2025-223014 online supplemental file 1

10.1136/thorax-2025-223014 online supplemental file 2

## Data Availability

Data are available upon reasonable request.
