# Supplementary material for: Systematic review and meta-analysis of the efficacy of biologic and targeted synthetic therapies in sarcoidosis
Source: Thorax. 2025 May 19;80(10):e223014. doi: 10.1136/thorax-2025-223014 (PMC12505053; doi:10.1136/thorax-2025-223014)
Supplement: online supplemental file 1 [file thorax-80-10-s001.docx]

Contents

[**Supplementary 1.** Summary of included comparator arm studies, with additional data column to table 1. 2](#_Toc197960295)

[**Supplementary 2a.** Risk-of-bias assessment of the included comparator studies using Version 2 of the Cochrane risk-of-bias tool for randomised trials (RoB 2). 5](#_Toc197960296)

[**Supplementary 2b**. Risk-of-bias assessment of the included single armed studies using the Newcastle Ottawa Scale (NOS). 6](#_Toc197960297)

[**Supplementary 3.** Vote counting data synthesis based on the direction of effect across outcomes, presented by drug, trial, and outcome 7](#_Toc197960298)

[**Supplementary 4.** Improvement in pulmonary function 10](#_Toc197960299)

[**Supplementary 5.** Leave one out analysis i) for all studies and ii) restricted to studies of anti-TNF class. 11](#_Toc197960300)

[**Supplementary 6**. Sensitivity analysis restricting to studies without high risk of bias 12](#_Toc197960301)

[**Supplementary 7**. Funnel plots, assessing potential publication bias in pairwise meta-analysis, primary outcome 13](#_Toc197960302)

| Supplementary 1. Summary of included comparator arm studies, with additional data column to table 1. | | | | | | | | | | | | | | |
| --- | --- | --- | --- | --- | --- | --- | --- | --- | --- | --- | --- | --- | --- | --- |
| Randomised control trials | | | | | | | | | | | | | | |
| Study | Design | Duration (weeks) | No. in study | Drug | No. on drug | Dose | Age | Female | Black | White | Duration disease | Concomitant medications | Predicted FVC | Primary Outcome |
| *Culver et al., 2023* | RCT | 24 | 37 | Efzofitimod | 9 | 5mg/kg | 50.8 ± 9.8 | 55.6 | 66.7 | 33.3 | 2.9 ± NR^ | CS (100), MTX (33.3), AZA (11.1) | 83.8 ± 16.6 | TEAE-free survival at wk 24 |
|  |  |  |  | Efzofitimod | 8 | 3mg/kg | 51.8 ± 11.4 | 50 | 25 | 75 | 4.3 ± NR^ | CS (100), LEF (12.5) | 83.8 ± 7.3 |  |
|  |  |  |  | Efzofitimod | 8 | 1mg/kg | 54.5 ± 11.3 | 50 | 37.5 | 62.5 | 5.3 ± NR^ | CS (100), MTX (25), HCQ (12.5) | 68.3 ± 9.7 |  |
|  |  |  |  | Placebo | 12 | NA | 52.5 ± 10.2 | 58.3 | 25 | 75 | 2.9 ± NR^ | CS (100), MTX (33.3), AZA (16.7) | 77.3 ± 11.5 |  |
| *Judson et al., 2014* | RCT | 28 | 173 | Ustekinumab | 60 | 180mg -> 90mg | 49.8 ± 10.2 | 48.3 | 31.7 | 63.3 | NR | CS (76.7), MTX (20), AZA (1.7) | 64.3 ± NR | Change at wk 16 in % predicted FVC |
|  |  |  |  | Golimumab | 55 | 200mg -> 100mg | 50.0 ± 9.4 | 49.1 | 29.1 | 65.5 | NR | CS (80), MTX (21.8), AZA (7.3) | 68.0 ± NR |  |
|  |  |  |  | Placebo | 58 | NA | 49.5 ± 9.5 | 50 | 39.7 | 55.2 | NR | CS (70.7), MTX (18.9), AZA (5.2) | 68.4 ± NR |  |
| *Pariser et al., 2013* | RCT | 12 | 15 | Adalimumab | 10 | 80mg -> 40mg | 46.0 ± NR | 80 | 100 | 0 | 9.8 ± NR | NR | NR | PGA score of 2 or less |
|  |  |  |  | Placebo | 5 | NA | 52.6 ± NR | 80 | 80 | 20 | 5.6 ± NR | NR | NR |  |
| *Baughman et al., 2006* | RCT | 24 | 138 | Infliximab | 47 | 5mg/kg | 46.5 ± 8.7 | 40.4 | 36.2 | 59.6 | 5.8 ± 6.1 | CS only (51.1), csDMARD only (8.5), CS+csDMARD (40.4) | 69.5 ± 8.6 | Change at wk 24 in % predicted FVC |
|  |  |  |  | infliximab | 46 | 3mg/kg | 49.3 ± 9.4 | 47.8 | 17.4 | 78.3 | 8.0 ± 6.2 | CS only (43.5), csDMARD only (8.7), CS+csDMARD (47.8) | 67.7 ± 9.6 |  |
|  |  |  |  | Placebo | 45 | NA | 45.3 ± 9.4 | 42.2 | 35.6 | 64.4 | 7.0 ± 6.2 | CS only (57.8), csDMARD only (4.4), CS+csDMARD (37.8) | 68.8 ± 11.1 |  |
| *Judson et al., 2008* | Analysis Baughman 2006 | 24 | 138 | Infliximab | 93 | 3mg/kg or 5mg/kg | 47.8 ± 9.1 | 44.1 | 26.9 | 68.8 | 6.9 ± 6.2 | NR | 68.6 ± 9.1 | Change at wk 24 in ePOST |
|  |  |  |  | Placebo | 45 | NA | 45.3 ± 9.4 | 42.2 | 35.6 | 64.4 | 7.0 ± 6.2 | NR | 68.8 ± 11.1 |  |
| *Baughman et al., 2005* | RCT | 26 | 18 | Etanercept | 9 | 25mg | NR | 88.9 | 77.8 | NR | NR | CS (55.6), MTX (100), AZA (0) | 68 ± NR | Ophthalmologist exam atr 6m |
|  |  |  |  | Placebo | 9 | NA | NR | 100 | 55.6 | NR | NR | CS (22.2), MTX (100), AZA (0) | 92 ± NR |  |
| *Rossman et al., 2006** | RCT | 6 | 19 | Infliximab | 13 | 5mg/kg | 46.77 ± 2.31 | 61.5 | 38.5 | 61.5 | NR | CS (69.2), MTX (NR), AZA (NR) | 50.6 ± 4.4 | Change at wk 6 in % predicted FVC |
|  |  |  |  | Placebo | 6 | NA | 49.33 ± 4.92 | 16.7 | 50 | 50 | NR | CS (66.7), MTX (NR), AZA (NR) | 56.8 ± 5.2 |  |
| *Kron et al., 2023* | RCT | 4 | 16 | Anakinra + SOC | 7 | 100mg per day | NR | NR | NR | NR | NR | NR | NR | Change in hs-CRP at 28 days |
|  |  |  |  | SOC | 9 | NR | NR | NR | NR | NR | NR | NR | NR | SOC |
| Single arm trials | | | | | | | | | | | | | | |
| Study | Design | Duration (weeks) | Total N | Drug | Drug  N | Dose | Age | Female | Black | White | Duration disease | Concomitant medications | Predicted FVC | Primary Outcome |
| *Friedman et al., 2021* | Open-label | 16 | 5 | Tofacitinib |  | 5mg BD | 40.8 ± NR | 20 | 20 | 80 | 2.75 ± NR | CS (100), MTX (NR), AZA (NR) | 86.6 ± NR | ≥ 50% reduction in CS at wk 16 |
| *Damsky et al., 2022* | Open-label | 26 | 10 | Tofacitinib |  | 5mg BD | 56 ± NR | 40 | 60 | 40 | 13.2 ± NR | CS (50), MTX (40), AZA (0), HCQ (10) | NR | Change in CSAMI activity score at 6 months |
| *Utz et al., 2003* | Open-label | 52 | 17 | Etanercept |  | 25mg twice weekly | 49.4 ± 10.7 | 58.8 | 11.8 | 88.2 | NR | NR | 91.1 ± 16.5 | NA |
| *Sweiss et al., 2014* | Open-label | 52 | 11 | Adalimumab |  | 40mg weekly | 45.3 ± 12.7 | 90.9 | 100 | 0 | NR | CS (45.5), MTX (36.4), AZA (9.1), LEF (9.1), MMF (36.4), CYC (9.1) | 61 ± 12 | Change from baseline to Week 24 in % predicted FVC |
| *Kullberg et al., 2020* | Open-label | 26 | 13 | Infliximab |  | 3-5mg | 47.6 ± 5.1 | 15.4 | 0 | 100 | 4.94 ± 5.0 | CS (92), MTX (16.7)^#^, AZA (0) | 70 ± NR | NA |
| *Vorselaars et al., 2015* | Open-label | 26 | 56 | Infliximab |  | 5mg/kg | 48.7 ± 10.1 | 35.7 | NR | 87.5 | 6.8 ± 7.1 | CS (42.9), MTX (82.1), AZA (7.1), LEF (1.8) | 78.8 ± NR | NA |
| *Sweiss et al., 2014* | Open-label | 52 | 10 | Rituximab |  | 1g weeks 0 and 2 | 49 ± NR^ | 30 | 30 | 60 | NR | NR | 57.3 ± 14.2 | Safety |
| *Baker et al., 2023** | Open-label prior to RCT | 16 | 15 | Sarilumab |  | 200mg | 57.0 ± NR^ | 20 | 20 | 73.3 | 5.17 ± NR^ | CS (100), DMARDs (33.3) | 92.0 ± NR^ | Flare-free survival on CS taper. Flare was defined as the need for rescue, significant worsening of disease, or cessation of study intervention. |

# **Supplementary 2a.** Risk-of-bias assessment of the included comparator studies using Version 2 of the Cochrane risk-of-bias tool for randomised trials (RoB 2).

| Bias | | | | | | |
| --- | --- | --- | --- | --- | --- | --- |
| Study | Randomisation Process | Deviations from intended interventions | Missing outcome data | Measurement of the outcome | Selection of the reported results | Overall |
| Culver, 2023 |  |  |  |  |  |  |
| Judson, 2014 |  |  |  |  |  |  |
| Parisier, 2013 |  |  |  |  |  |  |
| Baughman, 2006 |  |  |  |  |  |  |
| Baughman, 2005 |  |  |  |  |  |  |
| Rossman, 2006 |  |  |  |  |  |  |
| Baker, 2023 |  |  |  |  |  |  |
| Kron, 2023 |  |  |  |  |  |  |

Green, low risk of bias.

Yellow, some concerns of risk of bias.

Red, high risk of bias

Although Kron et al is not a randomised study, it was analysed using the RoB to allow comparison

# **Supplementary 2b**. Risk-of-bias assessment of the included single armed studies using the Newcastle Ottawa Scale (NOS).

| Bias | | | | | | |
| --- | --- | --- | --- | --- | --- | --- |
| Study | Representativeness of the cohort | Ascertainment of the exposure | Demonstration that the outcome of interest was not present at start of the study | Assessment of outcome | Was follow-up long enough for outcomes to occur | Adequacy of follow-up |
| Friedman et al., 2021 |  |  |  |  |  |  |
| Damsky et al., 2022 |  |  |  |  |  |  |
| Utz et al., 2003 |  |  |  |  |  |  |
| Sweiss et al., 2014a (adalimumab) |  |  |  |  |  |  |
| Kullberg et al., 2020 |  |  |  |  |  |  |
| Vorselaars et al., 2015 |  |  |  |  |  |  |
| Sweiss et al., 2014b (rituximab) |  |  |  |  |  |  |

Green, low risk of bias.

Yellow, some concerns of risk of bias.

Red, high risk of bias

Two NOS criteria were excluded as studies did not include a control arm; selection of the non-exposed cohort and comparability of cohorts.

# **Supplementary 3.** Vote counting data synthesis based on the direction of effect across outcomes, presented by drug, trial, and outcome

| **Authors** | **Study type** | **N** | **Pulmonary** | **Cutaneous** | **Cardiac/Ocular** | **e-POST** | **IS dose** | **18F-FDG-PET** | **PRO** |
| --- | --- | --- | --- | --- | --- | --- | --- | --- | --- |
| **Infliximab** |  |  |  |  |  |  |  |  |  |
| Rossman | RCT | 13 | VC: 15.2± 9.9, NS  CXR: 31%, NS  Dyspnoea: 0.38±0.21, NS |  |  |  |  |  | SF36: 0.39, NS |
| Baughman/  Judson | RCT | 93 | FVC: 2.5^, p=0.038  6WMD: 7.6±6.6  Borg's: 0.1±1.8, NS  CXR: 0.9±2.9, p=0.001 |  |  | 2.09±0.32, p=0.002 |  |  | SGRQ: 3.7±1.5, NS |
| Vorselaars | Single arm | 56 | PFT: 6.6^ | Skin lesion: 4 of 4 |  |  | Reduce GC: 8.8mg, p=0.001 | SUV 3.93, p<0.0001 | PGA: 8.2, p=0.009  SF36: 14.6, p<0.0001 |
| Kullberg | Single arm | 13 | FVC: 0.07 (-0.01 to 0.15)* |  |  |  | Reduce IS: 7 of 13 |  |  |
| **Adalimumab** |  |  |  |  |  |  |  |  |  |
| Sweiss | Single arm | 11 | FVC: 3 (-3 to 13)  6WMD: 20 (-90 to 124)*  Borg: 0.5 (0 to 1)*  CXR: 2 of 6 improved |  |  |  |  |  | PGA: 25 (10-42)* |
| Pariser | RCT | 10 | FVC: 0.09^ | PGA ≤2:OR 2.5(0.2,141)*  TL area: 32%, p=0.023  TL vol: 59%, NS |  |  |  |  | SHQ: 0.47  DLQI: 3.08, NS |
| **Etanercept** |  |  |  |  |  |  |  |  |  |
| Utz | Single arm | 17 | FVC: 4 of 17  CXR: 2 of 17  mMRC dyspnoea: 5 of 17 |  |  |  |  |  | SF36: NR, NS |
| Baughman | RCT | 9 |  |  | OGA: 2 of 9 |  | Reduce GC: 2 of 9 |  |  |
| **Golimumab** |  |  |  |  |  |  |  |  |  |
| Judson | RCT | 55 | FVC: 1.15 ± 1.41^, NS 6MWD: 12.5 ± 14.9^, NS | SPGA: 9 of 17  SASI: 2.57, NS  TL score: 2.3, NS |  | 3^, p=0.004 | Reduce GC: 34 of 42, p=0.01 |  | PGA: NR, NS  SAT: NR, NS  FAS: NR, NS  SGRQ: NR, NS  SF36: NR, NS |
| **Rituximab** |  |  |  |  |  |  |  |  |  |
| Sweiss | RCT | 10 | FVC: 2.9 (-4.9 to 14)*, NS  CXR: NR  6MWD: 19 (-38 to 80)* |  |  |  |  |  |  |
| **Ustekinumab** |  |  |  |  |  |  |  |  |  |
| Judson | RCT | 60 | FVC -0.15±1.30, NS  6MWD: -13.2±13.6, NS | sPGA: 3 of 21  TL score: 1.2, NS  SASI: 0.5, NS |  | 1^, NS | Reduce GC: 27 of 46, NS |  | PGA, NR, NS  SF36 , NR, NS  SAT, NR, NS  FAS, NR, NS  SGRQ, NR, NS |
| **Sarilumab** |  |  |  |  |  |  |  |  |  |
| Baker | RCT | 15 | FVC: -3 | SASI: 0 (-13 to 1)* |  | 0 |  |  | PGA: -2.5  HAQ: 0  FACIT-F: 1 |
| **Anakinra** |  |  |  |  |  |  |  |  |  |
| Kron | Single arm | 16 |  |  | LVEF: 5 (-3.4 to 14.7), NS |  |  | Cardiac PET: 17%, NS |  |
| **Efzofitimod** |  |  |  |  |  |  |  |  |  |
| Culver | RCT | 9 | FVC: 2.5^ |  |  |  | Reduce GC: 58.1% |  | SAT: 7.77, p=0.01  FAS: 16.17, p=0.022  KSQ: 6.42, p=0.018 |
| **Tofacitinib** |  |  |  |  |  |  |  |  |  |
| Friedman | RCT | 5 | FVC: 1^*  CXR: 2 of 5 |  |  |  | Reduce GC: 3 of 5 |  | SGRQ: 18.5 |
| Damsky | Single arm | 10 |  | CSAMI: 83% (40%–100%) |  |  | Reduce GC: 4 of 5 | 5 of 8 |  |

**Legend:**

Red box: unsupportive; Yellow box: Uncertain; Green box: supportive. FVC: % Force vital capacity ; CXR: Chest X-ray; 6MWD: 6-min walking distance ; Borg: Borg Dyspnea Scale; DLQI: Dermatology Life Quality Index questionnaire; CSAMI: Cutaneous Sarcoidosis Activity and Morphology Instrument; sGPA: Static Physician's Global Assessment; SASI: Sarcoidosis Activity and Severity Index; ePOST: extrapulmonary physician organ severity tool; IS: immunosuppression; GC: Glucocorticoid; PGA: Patient global assessment; HAQ: Health Assessment Questionnaire; FAS: Fatigue Assessment Scale; SHQ: Sarcoidosis Health Questionnaire; KSQ: King’s Sarcoidosis Questionnaire;

SAT: Sarcoidosis Assessment Tool; FACIT: Functional Assessment of Chronic Illness Therapy; SF36: 36-Item Short Form Health Survey; St Georges Respiratory Questionnaire.

Median and IQR presented unless specified. # = mean value presented with SD. * = value calculated using data in text, table or figure

P value = statistically significance when compared with placebo in RCT, and statistically significance when compared with baseline in single arm studies

For scores when a negative value indicates improvement, this was converted to a positive value e.g Borg Dyspnea Scale

**Outcomes and MCID:**

FVC: % Force vital capacity, MCID: 3-5.2% in Scleroderma (32), 2-6 in PFT (31)

6MWD: 6-min walking distance; MCID: 14-30.5m (41)

Borg Dyspnea Scale, range 0-10; MCID: 1 (42)

DLQI: Dermatology Life Quality Index questionnaire, range 0-30; MCID: 4 (43)

CSAMI: Cutaneous Sarcoidosis Activity and Morphology Instrument, range 0-165; MCID: 5 (44)

sGPA: Static Physician's Global Assessment; range 0-5 MCID: 2 (45)

SASI: Sarcoidosis Activity and Severity Index, range 0-72; MCID not established (46)

ePOST: extrapulmonary physician organ severity tool; range 0-102 MCID not established (47)

PGA: Patient global assessment; range 1-10, MCID: 2 (40)

HAQ: Health Assessment Questionnaire, range 0-3 MCID 0.25 (48)

FAS: Fatigue Assessment Scale; range 10-50, MCID:4 (49)

SHQ: Sarcoidosis Health Questionnaire, range 0-7; MCID not established (39)

KSQ: King’s Sarcoidosis Questionnaire; MCID: 8 (40)

SAT: Sarcoidosis Assessment Tool; range 29-203; MCID not established (50)

FACIT: Functional Assessment of Chronic Illness Therapy; range 0-52; MCID: 4 (51)

SF36: 36-Item Short Form Health Survey, range 0-100; MCID 2-4 (52)

St Georges Respiratory Questionnaire, range 0-100; MCID 5-8 (52)

|  | Study design | Study duration | Drug | Number on drug | Lung outcome | Mean or medium | SD or IQR or CI |
| --- | --- | --- | --- | --- | --- | --- | --- |
| *Baughman et al., 2006* | RCT | 24 | Infliximab | 47 | % change in FVC at 24 weeks | 2.5 | SD 0.7 |
| *Judson et al., 2014* | RCT | 28 | Ustekinumab | 60 | % change in FVC at 16 weeks | -0.15 | CI -2.68 to 2.38 |
| *Judson et al., 2014* | RCT | 28 | Golimumab | 55 | % change in FVC at 16 weeks | 1.15 | CI -1.64 to 3.95 |
| *Baker et al., 2023** | RCT | 16 | Sarilumab | 2 | % change in FVC at 16 weeks | -3.0^ | NR |
|  |  |  |  |  | Absolute change in FVC at 16 weeks | -110ml^ | NR |
| *Sweiss et al., 2014* | Open-label | 24 | Adalimumab | 11 | % change in FVC at 24 weeks | 3 | CI-3 to 13 |
| *Kullberg et al., 2020* | Open-label | 26 | Infliximab | 12 | % change in FVC at 26 weeks | 8~ | 11.8~ |
| *Vorselaars et al., 2015* | Open-label | 26 | Infliximab | 56 | % change in FVC at 26 weeks | 6.64 | 9.38* |
| *Sweiss et al., 2014* | Open-label | 24 | Rituximab | 10 | % change in FVC at 24 weeks | 6.61~ | 17.9~ |
| *Friedman et al., 2021* | Open-label | 16 | Tofacitinib | 5 | % change in FVC at 16 weeks | 1 | NR |
| *Rossman et al., 2006** | RCT | 6 | Infliximab | 13 | % change in FVC at 6 weeks | 15.22 | 9.91 |

# **Supplementary 4.** Improvement in pulmonary function

^median and not mean published

~ mean and SD calculated using published data.

* SD calculated using published data.

# **Supplementary 5.** Leave one out analysis i) for all studies and ii) restricted to studies of anti-TNF class.

# **Supplementary 6**. Sensitivity analysis restricting to studies without high risk of bias

# **Supplementary 7**. Funnel plots, assessing potential publication bias in pairwise meta-analysis, primary outcome
