# Supplementary material for: Systematic review and meta-analysis of the efficacy of biologic and targeted synthetic therapies in sarcoidosis
Source: Thorax. 2025 May 19;80(10):e223014. doi: 10.1136/thorax-2025-223014 (PMC12505053; doi:10.1136/thorax-2025-223014)
Supplement: online supplemental file 2 [file thorax-80-10-s002.pdf]

Systematic review and meta-analysis of the efficacy of biologic and targeted synthetic therapies in sarcoidosis

Bechman K et al. Thorax 2025. doi: 10.1136/thorax-2025-223014

Background: Infliximab is an established treatment for sarcoidosis. Trials have explored other biological and targeted synthetic therapies, but their clinical effectiveness is not clear.

Aim: To evaluate the role of biologic and targeted synthetic therapies in multi-system sarcoidosis

Methods: Meta-analyses were performed across multi-system outcomes, including lung function, skin scores, extrapulmonary organ severity tool (ePOST) and patient reported outcome measures (PROMs). A vote counting method was used to summarise estimates.

Meta-analyses also examined % predicted forced vital capacity (FVC), as mean change from baseline

Results:

Trial outcome data summarised using a traffic light matrix

	Study Type	Pulmonary	Cutaneous	Cardiac/Ocular	ePOST	Steroid Dose	PET-CT	PROM
Infliximab	RCT	Green						Yellow
	RCT	Yellow			Green			Yellow
	Single arm	Green	Green			Green	Green	Green
	Single arm	Yellow				Green		
Adalimumab	Single arm	Green						Green
	RCT	Yellow	Green					Green
Etanercept	Single arm	Red						Red
	RCT			Red		Red		
Golimumab	RCT	Yellow	Yellow		Green	Green		Red
Rituximab	RCT	Yellow						
Ustekinumab	RCT	Red	Red		Yellow	Green		Red
Sarilumab	RCT	Red	Red		Red			Red
Anakinra	Single arm			Yellow			Yellow	
Efzofitimod	RCT	Yellow				Green		Green
Tofacitinib	RCT	Yellow				Green		Green
	Single arm		Green			Green	Green	

Green: Supportive
Yellow: Uncertain
Red: Unsupportive

TNF inhibitors are associated with a modest improvement in FVC

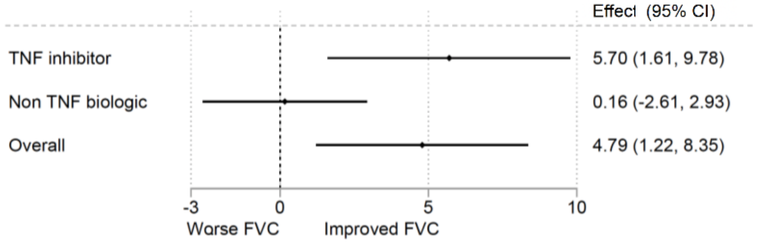

Key Messages: Evidence supports modest efficacy of infliximab for pulmonary sarcoidosis and promising but limited evidence for adalimumab, tofacitinib, efzofitimod. Larger, standardised trials are needed to refine treatment guidelines.
